# Assessing the Cost-Effectiveness of Photobiomodulation for Oral Mucositis Prevention and Treatment: A Systematic Review

**DOI:** 10.3390/biomedicines12102366

**Published:** 2024-10-16

**Authors:** Susell Parra-Rojas, Juliana Cassol Spanemberg, Nerea del Mar Díaz-Robayna, Mariela Peralta-Mamani, Rocío Trinidad Velázquez Cayón

**Affiliations:** 1 Oral Medicine and Phototherapy Research Group—OMEP, Faculty of Health Sciences, Department of Dentistry, Fernando Pessoa Canary Islands University, 35450 Las Palmas, Gran Canaria, Spain; sparra@ufpcanarias.es (S.P.-R.); rvelazquez@ufpcanarias.es (R.T.V.C.); 2 Faculty of Health Sciences, Department of Dentistry, Fernando Pessoa Canary Islands University, 35450 Las Palmas, Gran Canaria, Spain; nereadr95@outlook.es; 3 Hospital for Rehabilitation of Craniofacial Anomalies, University of São Paulo (HRAC-USP), Bauru 05508-020, Brazil; marielaperalta@alumni.usp.br

**Keywords:** oral mucositis, photobiomodulation, chemotherapy, radiotherapy, head and neck cancer

## Abstract

Background: We report on the cost-effectiveness of photobiomodulation (PBM) for the prevention and treatment of oral mucositis (OM) derived from the cytotoxic effects of antineoplastic therapy. Methods: This review followed the PRISMA 2020 guidelines. A search was conducted in PubMed, Scopus, Web of Science, Embase, and OpenGrey. Articles published before 23 July 2024, were included. Randomized controlled trials (RCTs) that included patients with head and neck cancer undergoing chemotherapy and/or radiotherapy and a placebo group compared to an intervention group (PBM) were selected. The risk of bias was evaluated using the Joanna Briggs Institute tools. The certainty of the evidence was assessed using the Grading of Recommendations Assessment, Development and Evaluation (GRADE) approach and was rated as moderate. Results: A total of 3 RCTs and 229 patients were included. PBM may represent an additional cost in the short term, but the incremental expenses derived from the cytotoxic effects of antineoplastic therapy are greater in the medium–long term. The intervention group (PBM) showed a lower incidence of severe OM compared to the control group (placebo). Conclusions: PBM is a cost-effective long-term treatment, effective in preventing severe OM and improving the quality of life of cancer patients. More RCTs following the same standardized protocols are needed (registration CDR42024498825).

## 1. Introduction

Oral mucositis (OM) induced by chemotherapy (CT) and radiotherapy (RT) is an inflammatory response of the oral tissues to the cytotoxic effect of the antineoplastic treatment on the mucosa. OM can occur in the oral cavity and throughout the gastrointestinal tract and is related to patient-inherent factors such as age, body mass index, oral cavity conditions, and genetic predisposition. Additionally, it is associated with the type, dose, and duration of CT and RT [1,2,3]. OM is a common side effect related to CT and RT, affecting 40% to 70% of patients. However, its incidence is higher in head and neck cancer (HNC) patients, reaching 85% to 90% of cases [1,2].

OM presents different clinical characteristics depending on its severity. Generally, it affects the oral mucosa and is accompanied by erythematous areas, pain, and/or ulceration, which may be accompanied by pseudomembranes. The most frequently affected areas are the buccal mucosa, tongue, lips, soft palate, and floor of the mouth. This condition significantly impacts conventional antineoplastic therapies because, in more severe cases, it leads to treatment interruption. Moreover, it is associated with increased costs due to a greater need for hospitalization, parenteral feeding, and higher medication consumption [4].

The main challenge faced by patients undergoing conventional antineoplastic treatment for head and neck cancer is the development of severe OM. This adverse effect is debilitating and affects the patient’s condition, the course of treatment, and additional costs [4,5,6].

OM significantly affects the patient’s daily life; once it develops, it inhibits eating, speaking, and drinking due to the intense pain experienced. This represents a major obstacle to treatment as it harms overall health, particularly nutritional status. Additionally, there is an increased need for hospitalization to manage both nutritional status and symptoms [1,6,7].

OM is estimated to be responsible for early treatment interruption in approximately 20% of cases, resulting in patients not receiving an optimal dose of CT, and this is associated with a reduced survival rate [8,9,10].

Evaluating the healthcare costs for an oncology patient is of great importance, as medical expenses grew exponentially, posing a significant challenge for the government. OM not only impacts the patient’s quality of life, but also leads to increased oncology therapy costs. These additional costs arise from the patient’s experience of intense pain, which increases the need for medication, hospital care, emergency visits, and auxiliary methods of feeding and hydration. The incremental costs associated with OM can range from USD 4.18 to USD 300,000, depending on the type of antineoplastic therapy, additional hospital expenses, the severity of OM, the resources used, and the country being evaluated. Therefore, it is crucial to find measures that mitigate the adverse effects of cancer treatment [4,10].

Photobiomodulation (PBM) is an alternative to conventional treatments (analgesics, opioids, mouth rinses, etc.) that proved promising in both the prevention and treatment of established OM [11,12,13,14,15,16,17,18,19,20,21,22]. Its mechanism of action is based on the use of low-power lasers with appropriate parameters to stimulate tissue repair, control inflammation, and alleviate pain [23,24,25,26,27]. Studies highlighted the need for standardization of dental treatment in OM patients [26], as the same low-power laser protocols and symptomatic treatments are not uniformly applied, although the use of red lasers with a wavelength of 630–660 nm stands out [25,26,27,28].

Subsequently, we aim to evaluate the cost-effectiveness of PBM to justify the additional costs of this treatment, which could, in turn, translate into reduced expenses related to hospitalization, nutritional support, and medication, among others. Numerous studies found that low-level laser therapy is cost-effective and efficient compared to other symptomatic treatments and provides a better quality of life for patients with OM [25,28]. Therefore, our objective is to report on the cost-effectiveness of PBM for the prevention and treatment of oral mucositis caused by the cytotoxic effects of antineoplastic treatment and to assess the efficacy of prevention protocols.

## 2. Materials and Methods

This systematic review is registered in the International Prospective Register of Systematic Reviews (PROSPERO) under the number CDR42024498825, following the Preferred Reporting Items for Systematic Reviews and Meta-Analysis (PRISMA) guidelines [29].

This systematic review aimed to answer the following question: in head and neck cancer patients treated with radiotherapy and/or chemotherapy, how effective and cost-effective is photobiomodulation in preventing and treating oral mucositis compared to placebo?

The PICO strategy was as follows:

P (patients): patients with head and neck cancer treated with radiotherapy and/or chemotherapy who developed oral mucositis.

I (intervention): photobiomodulation (PBM).

C (comparison): placebo (simulated PBM).

O (outcomes): efficacy and cost-effectiveness.

### 2.1. Eligibility Criteria

The selection criteria focused on a broad inclusion of articles, without restrictions on the year of publication or language. We selected only randomized clinical trials involving oncology patients that included prevention protocols with laser therapy to reduce the severity of OM and evaluated the cost-effectiveness of PBM. Additionally, the selected studies must assess the economic cost-effectiveness of PBM and compare a placebo group (simulated PBM) with a group that received actual PBM. We excluded all studies with only one group of patients (PBM without a control group), non-randomized clinical trials, cross-sectional studies, systematic reviews, observational studies, and letters to the editor.

### 2.2. Information Sources

An exhaustive search was conducted in the PubMed, Scopus, Web of Science (WoS), Embase, and OpenGrey databases. The search was updated to 23 July 2024. Additionally, the reference lists of the selected studies were manually reviewed to identify potential additional studies that met the inclusion criteria.

### 2.3. Search Strategy

The PICO strategy was used to define and guide the literature search to obtain results aligned with the research question. The descriptors were selected from the medical subject headings (MeSH) terms. The search strategy included relevant terms related to “oral mucositis”, “low-level laser therapy”, and “cost-effectiveness analysis”, along with their synonyms, using a combination of the Boolean operators “AND” and “OR” (Supplementary Materials S1).

### 2.4. Selection Process

The results were exported to the reference manager Zotero^®^ (v6.0.37, Zotero, version 6.0.37; Corporation for Digital Scholarship: Vienna, VA, USA, 2024) to exclude all duplicate studies. First, a selection was made based on the titles and abstracts of the articles found, eliminating those that did not meet the inclusion criteria. In the second phase, the presence or absence of a control group was confirmed, as well as whether the studies compared laser therapy use in patients with OM with other treatments and provided data on the analyzed variables. Two independent reviewers (N.D.R. and S.P.R.) participated in the selection process of the primary studies. Additionally, a third reviewer (J.C.S.) was included to resolve any discrepancies.

### 2.5. Data Collection Process and Data Items

Data collection was carried out using a pre-designed data extraction form. Two independent reviewers (N.D.R. and S.P.R.) collected data from the included studies, extracting the following information: author, year of publication, country, sample size, type of currency, type of healthcare system, type of cancer and antineoplastic treatment, photobiomodulation protocol, session duration, incidence of severe oral mucositis, other assessments (weight loss, use of a nasoenteral tube, need for gastrostomy for feeding), need for treatment interruption, cost analysis and analyzed variables, and results. Any discrepancies between the reviewers were resolved by a third reviewer (J.C.S.). Only the data available in the articles were obtained.

### 2.6. Study Risk of Bias Assessment

To assess the methodological quality of the studies included in this systematic review, the Joanna Briggs Institute (JBI) tool for randomized clinical trials was used [30]. This tool contains 13 questions that evaluate different aspects of the internal and external validity of the studies, including selection bias, intervention administration, outcome measurement, and participant retention. Each domain was independently evaluated by two reviewers, and discrepancies were resolved by a third reviewer. The questions addressed aspects such as the appropriate randomization of participants, allocation concealment, blinding of outcome assessors, and data integrity in the follow-up. Responses were categorized as “Yes”, “No”, or “Unclear”. Each evaluated domain was assigned a score, where 1 point indicated low risk, 2 points represented moderate risk, and 3 points indicated high risk. The total score for each study was obtained by summing the points from the 13 domains, and the studies were classified into three categories: low risk of bias (13–20 points), moderate risk of bias (21–26 points), and high risk of bias (>26 points). The evaluation results informed the analysis of the methodological quality of the studies and the interpretation of the findings.

### 2.7. Effect Measures

To evaluate the effects of photobiomodulation (PBM) in the prevention and treatment of oral mucositis induced by antineoplastic treatments in head and neck cancer patients, the incidence and severity of oral mucositis were reported using the World Health Organization Oral Mucositis Grading Scale, which classifies mucositis into four severity grades (I to IV). The reduction in the incidence of severe oral mucositis (grade III/IV) in the groups receiving PBM was measured compared to the control group (placebo), with results expressed in absolute values and percentages.

The economic cost-effectiveness analysis included costs associated with the treatment of mucositis, such as hospitalizations, use of analgesics, nutritional support needs, interruptions in oncological treatment, and overall healthcare-related expenses. Cost data were standardized in dollars (USD), converting amounts when studies reported results in other currencies. Economic results were also expressed in absolute values and percentages to facilitate comparisons between studies.

### 2.8. Synthesis Methods

To synthesize the results from the included studies, a narrative analysis was conducted as the heterogeneity among the studies regarding interventions, outcomes, and methodologies did not allow for a meta-analysis. The studies were grouped based on patient characteristics, the type of antineoplastic treatment received (chemoradiotherapy), the use of photobiomodulation (PBM), and the reported outcome measures, such as the reduction in the severity of oral mucositis and the costs associated with treatment.

The qualitative synthesis was based on comparing the effectiveness of PBM in preventing severe oral mucositis (grade III/IV) and the economic cost-effectiveness analyses, considering both direct and indirect costs (hospitalizations, use of analgesics, etc.). Studies that reported financial data in different currencies were converted to U.S. dollars to facilitate comparison.

### 2.9. Certainty Assessment

The assessment of the certainty of evidence in the included studies was conducted using the Grading of Recommendations, Assessment, Development, and Evaluations (GRADE) tool. This method allowed us to evaluate the quality of evidence based on the following domains: risk of bias, inconsistency, indirectness, imprecision, and publication bias.

## 3. Results

### 3.1. Study Selection

The study selection process followed the PRISMA 2020 guidelines [29], utilizing multiple databases and registries, as well as additional sources. A total of 248 studies were identified. After the removal of 86 duplicate records, 162 studies remained for the first selection phase. Following the reading of titles and abstracts, 157 studies were excluded as not meeting the inclusion criteria. Of the five remaining studies from the databases and gray literature, full articles were evaluated, resulting in the exclusion of one study for lacking a control group and one study for not assessing cost-effectiveness.

**Figure 1:**
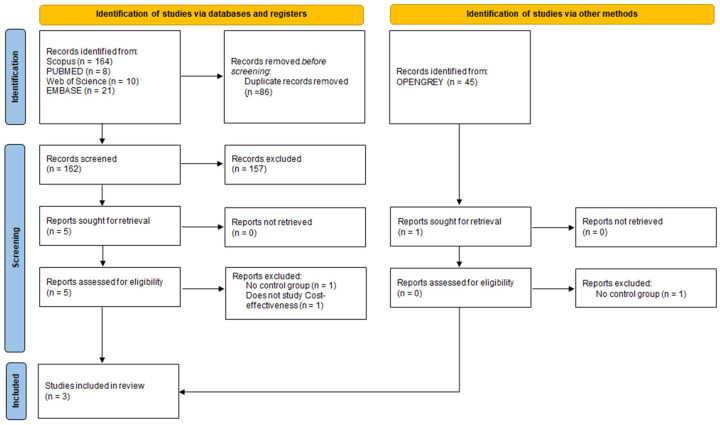
The flowchart of the included studies.

Finally, three studies met the inclusion criteria and were selected for this systematic review: Nugent et al. [31], Lopes Martins et al. [32], and Antunes et al. [33]. The screening and inclusion stages were reported following the PRISMA flow diagram (Figure 1).

### 3.2. Study Characteristics

Three articles were selected with a total sample of 229 patients: 192 men and 37 women, with a mean age of 58 years (ranging from 40 to 75 years). The geographical context of the studies varied, although all were within the public healthcare system. The study by Nugent et al. [31] was conducted in England, Scotland, and Wales, while the studies by Lopes Martins et al. [32] and Antunes et al. [33] took place in Brazil.

The studies included patients with squamous cell carcinoma. The initial tumor locations in head and neck cancer patients varied across different anatomical areas. In the Nugent et al. [31] study, most tumors were located in the oropharynx (67 patients), with additional cases in the oral cavity (11), larynx (3), and nasopharynx (3). In the Lopes Martins et al. [32] study, the main locations were the base of the tongue (26 patients), followed by the tongue (7), hypopharynx (5), glottis/supraglottis (4), floor of the mouth (2), oral mucosa (2), and hard palate (1). Finally, in the Antunes et al. [33] study, the oropharynx was the predominant location (74 patients), followed by the hypopharynx (11) and the nasopharynx (9).

The treatment received by the patients for their cancer type consisted of radiotherapy (RT) or a combination of chemoradiotherapy (CRT). Patients in the Nugent et al. [31] study received RT, with a minimum dose of 60 Gy. In the Lopes Martins et al. [32] study, patients received a minimum RT dose of 50 Gy, with or without chemotherapy (QT). However, in the Antunes et al. [33] study, patients had a prescribed RT of 1.8 Gy/day (a total of 70.2 Gy) for 5 days a week, with concomitant cisplatin chemotherapy (QT) of 100 mg/cm^2^ every 3 weeks.

All studies included two groups: an intervention group that received photobiomodulation (PBM) and a control group that received a simulation of PBM (placebo). Both groups followed adjunctive measures, such as the use of medication, fluoride rinses, chlorhexidine rinses, and oral hygiene recommendations for the management of oral mucositis.

The type of currency used also varied. Nugent et al. [31] utilized British pounds, while both Brazilian studies, Lopes Martins et al. [32] and Antunes et al. [33], converted Brazilian reals to U.S. dollars. To standardize costs and allow for a more accurate comparison between studies, all currencies were converted to U.S. dollars. It is important to note that monetary values may have changed since the studies were conducted, and as of 30 March 2024, GBP 1 is equivalent to USD 1.25 (Table 1, Table 2 and Table 3).

### 3.3. Risk of Bias in Studies

**Figure 2:**
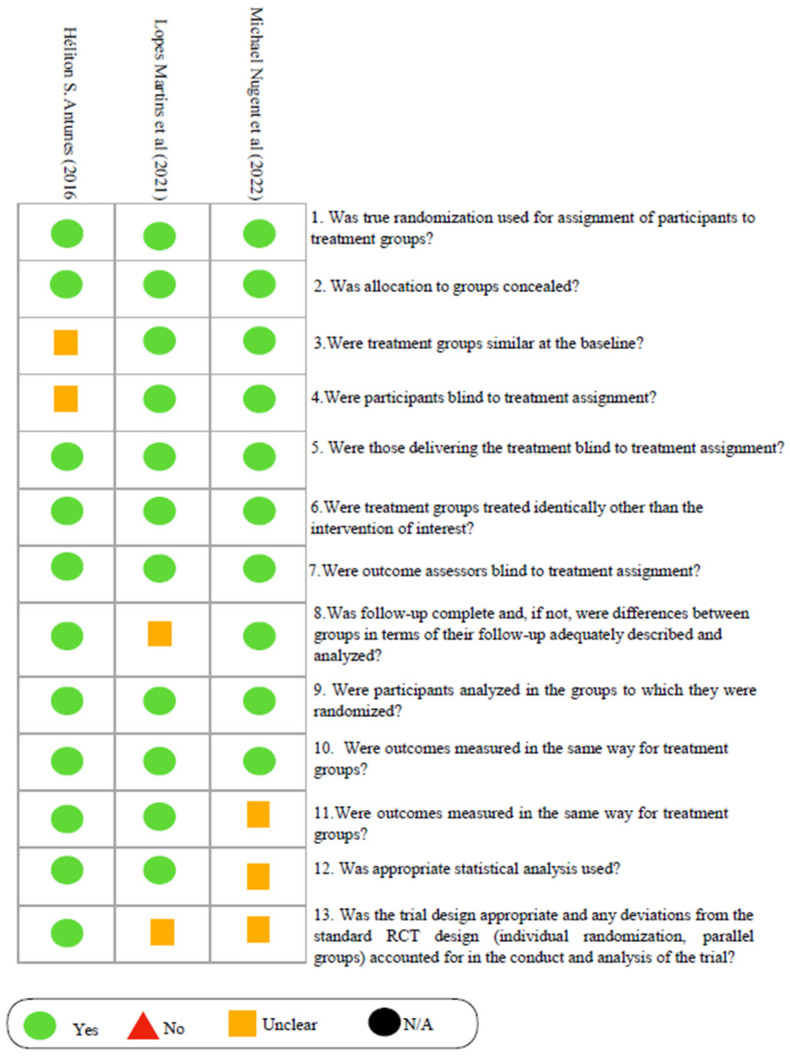
Risk of bias in the studies, “N/A” stands for “Not Applicable” and is used when specific information is not relevant or available for the context.

The three studies included in this systematic review presented a low risk of bias, according to the evaluation based on key domains such as randomization, blinding, and handling of incomplete data. Antunes et al. [33] achieved a total score of 17, with minor concerns regarding the management of incomplete data, but with adequate controls in randomization and blinding. López Martins et al. [32], with a score of 15, also showed a low risk of bias, although there were some concerns regarding allocation concealment. Meanwhile, Nugent et al. [31], with a score of 14, was also classified as having a low risk of bias, despite slight concerns about data follow-up (Figure 2).

### 3.4. Results of the Individual Studies

The laser protocol received by the intervention group varied in each trial. Nugent et al. [31] scheduled three sessions per week for 6 weeks prior to the radiation therapy (RT) sessions. A diode laser (660 nm) was used, with an output power of 74 mW, an area of 1.5 cm^2^, an irradiance of 50 mW/cm^2^, an exposure time of 60 s, and a fluence of 3 J/cm^2^. The estimated time required for each session ranged between 20 and 30 min. Lopes Martins et al. [32] planned five laser sessions per week between RT sessions. A diode laser (660 nm) was employed with a power of 25 mW and energy delivered per point of 0.25 J, totaling 6.2 J/cm^2^. The total energy applied daily was 15.25 J. Antunes et al. [33] organized a daily preventive laser session before the application of radiotherapy. They used a diode laser (660 nm) with a power of 100 mW in a 0.24 cm^2^ area, delivering 4 J/cm^2^. The estimated time per laser session was 12 min per patient.

Regarding the reduction in the incidence of severe oral mucositis (OM), defined as OM of grade III/IV according to the WHO, Nugent et al. [31] determined that after 6 weeks of the intervention, there was an increase in severe OM in 10% of patients in the control group, while at 4 months, 3.2% of patients in the laser group (1 out of 44) experienced severe OM compared to 2.9% of the control group (1 out of 43). Lopes Martins et al. [32] demonstrated that the incidence of severe OM was lower in the intervention group, where 16 patients did not present severe OM, compared to 8 patients in the control group who also did not present it. Additionally, the group that received photobiomodulation (PBM) showed a lower incidence of severe OM in sessions 21 and 30 of radiation therapy. Antunes et al. [33] established that the highest incidence of severe OM was in the placebo group, as 19 out of 47 patients developed it, compared to 3 out of 47 patients in the laser group.

The additional aspects evaluated varied among the studies. Nugent et al. [31] assessed weight loss and body mass, oral intake, and dependency on a feeding tube, in addition to the use of analgesics. These parameters were evaluated at 6 weeks and 4 months. At 6 weeks, in the intervention group, six patients lost more than 10% of their weight, indicating the need for nutritional support. In contrast, only two patients in the control group experienced weight loss greater than 10%.

However, at 4 months, the situation changed: the percentage of patients with more than 10% weight loss was higher in the control group, with 17 out of 44 patients. Regarding the need for a feeding tube, at 6 weeks, the proportion of patients was equal in both groups. Nevertheless, at 4 months, 11 out of 44 patients in the laser group retained the feeding tube, while only 6 patients in the control group retained it.

In this regard, Lopes Martins et al. [32] reported that only two patients in the PBM group required a nasoenteral tube, compared to four in the control group. Meanwhile, Antunes et al. [33] found that the placebo group had a greater need for gastrostomy as a means of feeding, with a total of 18 out of 47 patients, in contrast to 7 out of 47 patients in the laser group.

Regarding the use of analgesics, Nugent et al. [31], Lopes Martins et al. [32], and Antunes et al. [33] indicated that the highest analgesic use was in the control group. The analgesics used in the studies ranged from acetaminophen and non-steroidal anti-inflammatory drugs (NSAIDs) to opioids.

The need for treatment interruption, according to Nugent et al. [31], affected a total of 18 patients, of which 6 were from the intervention group and 12 from the control group. In the intervention group, treatment was interrupted between the second and fifth week, with at most two laser sessions received and, in one case, three sessions. Lopes Martins et al. [32] indicated that RT was interrupted in nine patients from the control group, compared to two from the intervention group. In the laser group, treatment was interrupted in the second and third weeks, while in the control group, it was interrupted in the second, third, and last weeks. Antunes et al. [33] did not report these data.

Nugent et al. [31] concluded that their economic evaluation was compromised by the sample size, as well as the estimation of the cost of laser sessions per patient. They determined that this expense amounted to USD 1012.6 per patient for 18 sessions. They estimated that hospitalization costs between weeks 2 and 6 were nearly equal for the laser group and the control group, with figures of USD 2039.10 and USD 2036.57, respectively. However, this changed between the sixth week and the fourth month, as the expenses were higher in the control group at USD 1789.10, compared to USD 1112.35 for the intervention group.

On the other hand, higher primary care and outpatient costs were assessed in the control group, with figures of USD 189.39 and USD 789.12, respectively. These figures totaled USD 135 in primary care and USD 666.65 in outpatient costs for the laser group. In terms of medication expenses, estimated costs were relatively higher in the laser group at USD 358.58, while the control group had medication costs of USD 273.98.

Lopes Martins et al. [32] concluded that the results favor the integration of laser therapy for the prevention and treatment of severe oral mucositis associated with the toxicity of treatment for head and neck cancer. Additionally, integration improved the quality of life of patients concerning their oral health and reduced the need for interruptions in radiotherapy (RT). These authors estimated that the average cost of laser treatment per patient for 34 sessions was approximately USD 900.16, considering various associated expenses such as equipment acquisition, consumables, services, etc. Therefore, the use of laser therapy in the intervention group had an incremental cost of USD 857.35. In terms of additional interventions, medication, nasoenteral tubes, and electrolyte replacement agents and multivitamins, the expenses were higher for the control group, with an average cost of USD 185.17, while for the laser group, the average cost was USD 27.44.

Antunes et al. [33] found that, despite its increased cost of treatment, laser therapy proved effective in reducing the incidence of severe oral mucositis associated with cancer treatment and the costs associated with it. They set the incremental cost at USD 251.14 per patient for the control group, while for the intervention group, it was USD 59.57. This incremental cost did not account for the expenses associated with laser therapy, but did consider hospitalization costs, the use of opioids, and the need for gastrostomy. Furthermore, they estimated the increase in cost with the use of laser therapy as USD 1689 per patient. In conclusion, these authors observed the economic impact of the treatment but also acknowledged the advantages of laser therapy in reducing the incidence of severe oral mucositis and the associated costs.

When the three studies evaluated the cost-effectiveness of laser therapy, they all concluded that there was an increase in treatment costs of approximately USD 800 to USD 1700 [32,33]. However, benefits were also observed, such as a reduction in the costs associated with the toxicity of oral mucositis resulting from cancer treatment, a decrease in the incidence of severe oral mucositis, and a reduction in the need to interrupt cancer treatment [31,32,33]. This translates into an improvement in the quality of the patient’s life related to oral health. Lopes Martins et al. [32] noted that the impact profile on oral health was better in the laser group by week 30, as they obtained a statistically significant difference in their evaluation. However, when they assessed the symptoms of oral mucositis reported by patients, no statistical difference was perceived between the two groups. In fact, the total variation in oral mucositis symptoms was 11 in the laser group compared to 38 in the control group.

### 3.5. Certainty of Evidence

Based on the analysis of the certainty of evidence using the GRADE approach conducted with the GRADEpro GDT (version 2023; Evidence Prime: Hamilton, ON, Canada, 2023) tool, the results for both the cost-effectiveness analysis and the reduction in the incidence of severe oral mucositis were rated as moderate certainty. This assessment was primarily influenced by the small sample sizes in the included studies, which introduced a degree of imprecision. However, the risk of bias, inconsistency, and indirectness of the evidence were considered non-serious, supporting the overall moderate confidence in the findings. These results are considered critical for evaluating the clinical effectiveness and economic viability of PBM in the treatment of oral mucositis (Table 4).

## 4. Discussion

This systematic review is based on the high incidence of oral mucositis (OM) and the need for treatments that improve the quality of life for oncology patients while demonstrating economic viability. Currently, there are few studies on the cost-effectiveness of laser therapy, but there is considerable research on its therapeutic efficacy. This highlights the necessity of evaluating the cost-effectiveness of laser therapy to allow oncology units to acquire this technology and patients to benefit from its advantages. It is essential to consider the drastic changes that oncology patients experience owing to the side effects of cancer treatment and its cytotoxic effects. An example is the high prevalence of OM in patients with head and neck cancer [4,34,35,36].

The total sample studied in this systematic review consisted of 229 patients, of whom 116 received laser therapy as a method for preventing or treating severe OM, while 113 received a simulation of laser therapy. The number of participants is approximate, as the study by Nugent et al. [31] experienced patient dropouts as the study progressed.

Currently, cytotoxic effects occur due to both chemotherapy (CT) and radiotherapy (RT). No treatment acts solely on cancer cells, which often leads to side effects. OM is among the most debilitating adverse effects of conventional cancer treatment. Its incidence is particularly high in head and neck cancer patients undergoing CT, RT, or both. In the case of CT, the development of OM is associated with the drug used, the dosage, and the duration of treatment. The agents most frequently associated with OM include 5-fluorouracil (5-FU), platinum derivatives (cisplatin and oxaliplatin), carmustine, etoposide, cytarabine, and melphalan. However, there is no specific CT protocol linked to a higher incidence of OM. Regarding RT, the development of OM is related to radiation dose, irradiated tissue volume, fractionation schedule, and the simultaneous use of CT [37,38].

OM is an amplified inflammatory response produced by conventional antineoplastic treatment. This condition significantly impacts the patient’s quality of life, as in the most severe cases, it can lead to treatment interruption and an increased need for hospitalization, parenteral nutrition, and higher medication consumption [4,32,33].

According to the latest MASCC/ISOO update of the clinical practice guidelines, oral care protocols, anti-inflammatories, coating agents, anesthetics, analgesics, and photobiomodulation (PBM) are recommended for the management of oral mucositis (OM). In the case of radiotherapy (RT), it is recommended that PBM be applied throughout the entire course of treatment with a wavelength of 632.8 nm, an irradiance of 24 mW/cm^2^, and a fluence of 3 J/cm^2^ for 125 s over an area of 1 cm^2^, with a total of 12 points. However, this recommendation changes when RT is combined with chemotherapy (CT). It is maintained that PBM should be applied throughout the entire course of RT. The wavelength should be 660 nm, with an irradiance of 417 or 625 W/cm^2^ and a fluence of 4.2 J/cm^2^ or 6.2 J/cm^2^ for 10 s over an area of 0.24 or 0.04 cm^2^, with a total of 72 or 69 points [5].

In the studies selected for this review, different doses of RT were used, and only one study combined RT with CT, using cisplatin 100 mg/cm^2^ every 3 weeks. A total of 229 patients with head and neck cancer were analyzed, being divided into two groups: one group received PBM as a preventive method for OM before RT sessions, while the other group received a laser simulation along with conventional oral care treatment with medication. The laser protocols used in the studies were diverse.

The studies agreed on the wavelength, with the most suitable for treating oral mucosa lesions being red (660 nm). The choice of 660 nm light is based on its absorption by key cellular chromophores, particularly cytochrome c oxidase, which plays a crucial role in the mitochondrial respiratory chain. This absorption enhances adenosine triphosphate (ATP) production, providing cells with more energy to promote tissue repair. Additionally, red light is well absorbed by hemoglobin, making it particularly effective in highly vascularized tissues such as the oral mucosa. The increased cellular energy helps reduce inflammation by lowering the production of pro-inflammatory cytokines, which is critical for conditions such as oral mucositis (OM). This combination of improved energy metabolism and anti-inflammatory effects is central to the therapeutic benefits of 660 nm light in PBM [39,40,41].

However, the irradiance, fluence, application time, and number of points were different [31,32,33]. Other studies, such as those by Angélica F Oton-Leite et al. [40] and Vivian Youssef Khouri et al. [41], can corroborate this.

Of the total patients, 116 belonged to the laser group and 113 to the control group. The incidence of OM was higher in the control group, where 55 patients developed severe OM. However, this incidence was highest in the group that received 60 Gy of RT comprising 21 patients, followed by the group that received the combination of RT and CT. It is important to highlight that in the study by Nugent et al. [31], the laser was applied in the control group once the patients developed severe OM, which possibly altered the final results. According to the results obtained in the studies we analyzed, there was no greater incidence of OM when combining CT and RT, although there are many studies in which a higher incidence of OM was observed [31,32,33,37,38].

Of the 229 patients evaluated, 29 had to interrupt their cancer treatment, with the control group showing a higher number of interruptions, totaling 21 patients. This suggests that the preventive use of laser therapy possibly contributed to reducing the interruptions in anticancer treatment due to the lower incidence of severe OM [14,15,16,17,18]. However, owing to the lack of standardized protocols in the RCTs studied in this review, there was a notable heterogeneity in the data found, particularly concerning the laser protocols, which complicated the comparison of information. It can only be concluded that low-level laser therapy is effective when using a wavelength of 660 nm [14,32,33].

Notably, the cost of laser therapy does not represent an incremental expense in the long term because of the lower additional spending on medication, hospitalization, and treatment interruptions [14,32]. The implementation of laser therapy only requires an initial investment for the acquisition of the device, estimated at about USD 6000.00, according to Lopes Martins et al. [32]. In the article by Antunes et al. [33], a distinction is made between the incremental costs of laser therapy and without it; the authors concluded that laser therapy had an economic impact on treatment costs, as the intervention group incurred expenses of USD 1689, compared to USD 251.14 in incremental expenses for the control group. However, the incremental costs of anticancer therapy for head and neck cancer associated with a higher incidence of severe OM ranged from USD 1700.00 to USD 40,000.00 [32]. This increase is due to a greater need for medications, hospitalization, parenteral nutrition, and primary care. The analyzed studies concluded that these incremental costs were higher in the group that did not receive laser therapy in addition to anticancer treatment [14,32]. PBM parameters varied significantly across the three selected studies, including differences in wavelength, energy density, and application time. The cost-effectiveness analysis did not establish a single set of optimized PBM parameters due to this variability. This inconsistency poses a challenge when attempting to develop a standardized, cost-effective protocol from the available data. However, while all studies demonstrated the effectiveness of PBM in reducing severe OM, none provided enough consistent evidence to determine which specific parameters are the most cost-effective. Further research, potentially including a meta-analysis or randomized trials focused on parameter optimization, would be necessary to identify an ideal set of PBM parameters for both clinical effectiveness and cost-efficiency. Therefore, considering these aspects, the introduction of photobiomodulation is deemed more cost-effective, not only due to its economic feasibility, but also because of the implications for the patient in avoiding severe OM and its consequences.

The timing of application and the number of laser sessions applied are of utmost importance, due to both the economic implications and the efficient allocation of additional expenses that laser therapy may incur in the short term. The number of laser sessions applied as a preventive measure against the development of severe oral mucositis (OM) was influential in the laser groups that received either three or five sessions before radiation therapy (RT). However, there were no notable differences between the groups that received five sessions during RT and those who received one session just before RT. Thus, according to the evidence presented, we can conclude that the number of laser sessions does influence the prevention of severe OM, but the timing of application is not of great relevance.

In contrast to the previously presented data, Oliveira FM et al. [42] demonstrated the effects of a difference in the timing of laser application, as the group that received laser sessions on alternate days had a higher incidence of OM compared to the group that received them on consecutive days. Considering the data provided by Antunes et al. [33], applying the laser before RT is sufficient, results in a lower incremental expense for laser therapy than if five sessions are applied within a week. This would lead to better management of economic resources and a lower incidence of severe OM, which is essential for the patient’s quality of life [31,32,33,42].

The consequences of severe OM in oncology patients are varied. They can range from poor nutrition and infections of ulcerative lesions to a higher mortality rate, which is estimated to be between 6% and 26% among patients. For this reason, managing and controlling this side effect is crucial. Additionally, malnutrition in these patients is directly associated with treatment interruptions, as undernourished patients tolerate treatment less well [43].

It is important to highlight the differences and similarities in oncology services in the area of dentistry in the countries studied in the clinical trials: Brazil and England. This allows us to compare the dental care that oncology patients receive in Spain, emphasizing the necessity of a multidisciplinary team to address all the needs of this type of patient. In Brazil, dental services are part of primary care in the Unified Health System (SUS). The national policy of oral health, also known as Brasil Sorridente (https://www.gov.br/saude/pt-br/composicao/saps/brasil-sorridente, accessed on 28 September 2024), was implemented in 2004. Oral health was designated as one of the four priority areas of the SUS, transforming oral health care in this country with the objective that the SUS achieve the integrality of care envisaged at its creation [44].

The costs of applying PBM vary significantly across regions and countries, and this variability can indeed impact the overall cost-effectiveness of the therapy. Factors such as healthcare infrastructure, equipment procurement, training, labor costs, and national healthcare policies all contribute to these regional differences. For instance, in countries such as Brazil, where public healthcare integrates dental services into oncology care, the costs of implementing PBM may be more manageable due to centralized purchasing and shared healthcare resources. In contrast, in countries with higher healthcare expenses, such as England, initial investments in equipment and higher operational costs might make PBM less immediately cost-effective unless supported by institutional funding or public health initiatives. Additionally, in low-resource settings, access to PBM technology could be limited, further affecting its cost-effectiveness. Future research and cost-effectiveness models should consider regional variations in cost structures when evaluating the broader application of PBM.

The integration of dental services into hospital centers significantly enhances patient access to care and fosters collaboration among healthcare professionals. This setup allows oncologists and dentists to work together as a multidisciplinary team, ensuring a holistic approach to patient care. By viewing the patient from multiple perspectives, the team can better address the complex needs of oncology patients, improving the coordination of treatments and overall outcomes. This model underscores the importance of integrated care in promoting both efficiency and comprehensive health management.

### Limitations

Among the limitations of this systematic review is the absence of standardized protocols regarding the low-intensity laser parameters used in the included studies. While all three studies analyzed employed laser therapy as a preventive method for the development of severe oral mucositis, none utilized the same parameters for irradiance, fluence, duration, or application points, despite using the same wavelength. This variation complicates the comparison and synthesis of results, though the effectiveness of intraoral red laser in preventing severe OM is evident.

Another notable limitation is the lack of clinical trials in the literature that objectively assess the cost-effectiveness of low-intensity laser therapy. This highlights a clear need for well-designed studies with larger sample sizes, capable of monitoring a greater number of cases. Furthermore, these studies should consider additional factors potentially influencing OM development, such as body mass index, pre-existing oral conditions, lifestyle factors, and toxic habits (e.g., tobacco and alcohol consumption).

Therefore, new randomized clinical trials with larger sample sizes and standardized protocols are necessary to further validate and solidify the conclusions of this review. Additionally, future studies that comprehensively evaluate the cost-effectiveness of photobiomodulation in different healthcare contexts would strengthen the body of evidence supporting its use. These efforts should encourage clinicians to design studies that assess PBM’s full potential in improving patient outcomes, focusing specifically on its effectiveness in reducing therapy-related adverse effects such as OM, rather than solely on the efficacy of primary treatments. By refining research designs and including cost-effectiveness evaluations, future investigations can significantly contribute to the establishment of PBM as a viable intervention for the prevention of severe OM and other treatment-related adverse effects.

## 5. Conclusions

Photobiomodulation is a promising and cost-effective intervention for reducing the incidence of severe oral mucositis in patients undergoing treatment for head and neck cancer. While initial costs may pose challenges, the long-term benefits, such as fewer hospitalizations, reduced need for medication, and overall improvements in patient quality of life, support its economic viability. However, despite its demonstrated effectiveness, caution is warranted, owing to the moderate certainty of the current evidence and the lack of standardized PBM protocols. Current studies suggest the efficacy of PBM for attenuating the adverse effects of cancer treatment, but uncertainties remain regarding specific parameters, such as irradiance and fluence. More studies are needed to fully validate photobiomodulation’s cost-effectiveness across various cancer-related toxicities and to encourage broader adoption in clinical settings.

## Figures and Tables

**Table 1:** Characteristics of the studies, type of currency used, and type of healthcare system.

Author	Country	Sample (*n*)	Healthcare System	Tumor Location and Prescribed Treatment	Photobiomodulation Protocol	Time per Session
Nugent et al., 2022 [31]	England, Scotland, and Wales	87 LG *n* = 44 CG *n* = 43	Public Healthcare System	Nasopharynx: 3, Oropharynx: 67, Larynx: 3, Oral cavity: 11, Unknown: 3 RT alone: 20, RT + CT: 67 RT with a minimum of 60 Gy.	• 3 sessions per week for 6 weeks • Diode laser (660 nm), power of 74 mW • Beam area of 1.5 cm^2^, irradiance 50 nW/cm^2^ • Exposure time of 60 s, fluence of 3 J/cm^2^ PBM was applied before RT sessions.	20–30 min
Lopes Martins et al., 2021 [32]	Brazil	48 LG *n* = 25 CG *n* = 23	Public Healthcare System	Oral mucosa: 2, Hard palate: 1, Tongue: 7, Floor of the mouth: 2, Tongue base: 26, Rhinopharynx: 1, Hypopharynx: 5, Glottis and supraglottis: 4 Conventional RT with a minimum dose of 50 Gy 63.88 (±14.24) + CT	• 5 sessions per week between RT sessions • Diode laser (660 nm), power of 25 mW • Energy deposited 0.25 J per point, 6.2 J/cm^2^ for 10 s Total daily energy applied 15.25 J.	Not specified
Antunes et al., 2016 [33]	Brazil	94 LG *n* = 47 CG *n* = 47	Public Healthcare System	Nasopharynx 9, Oropharynx 74, Hypopharynx 11 RT: 70, 2 Gy + CT concurrent cisplatin 100 mg/cm^2^ every 3 weeks.	• One daily preventive application before RT Diode laser (660 nm), power of 100 mW, in an area of 0.24 cm^2^ depositing 4 J/cm^2^.	12 min

Abbreviations: LG (laser group), CG (control group), RT (radiotherapy), CT (chemotherapy), Gy (gray), mW (milliwatts), nm (nanometers), and J (joule) cm (centimeter), PBM (Photobiomodulation).

**Table 2:** Incidence of oral mucositis and analyzed variables.

Author	Incidence of Severe OM	Additional Evaluations Assessed	Need for Treatment Interruption
Nugent et al. [31]	6 weeks CG (21 of 43 patients) LG (19 of 44 patients) 4 months CG (1 of 43) LG (1 of 44)	At 6 weeks: Greater weight loss >10% in the laser group, requiring nutritional support. = proportionate use of feeding tubes. At 4 months: Greater weight loss >10% in the control group. Increased use of feeding tubes in the laser group. Higher use of analgesics in the control group.	In total, 18 patients interrupted treatment before completing the 18 sessions outlined in the trial. Greater treatment interruption in the control group (12 patients).
Lopes Matins et al. [32]	CG (15 of 23) LG (9 of 25)	Greater need for nasoenteral feeding tube in the control group (4 out of 23 patients). Greater use of analgesics in the control group.	Greater treatment interruption in the control group (9 patients).
Antunes et al. [33]	CG (19 of 47) LG (3 of 47)	Greater need for gastrostomy feeding in the control group (18 out of 47 patients). Greater use of analgesics in the control group.	Not specified

Abbreviations: LG (laser group), CG (control group), OM (oral mucositis).

**Table 3:** Characteristics of reported currency, cost analysis, and main results.

Author	Currency Type (2024 USD)	Cost Analysis	Results
Nugent et al. [31]	Pounds (converted to USD)	Cost of laser sessions per patient: USD 1012.6 (18 sessions) • Hospitalization costs for LG: USD 2039.10 (between weeks 2 and 6). • Hospitalization costs for CG: USD 1789.10 (between week 6 and 4 months). • Higher outpatient costs for CG: USD 789.12 (evaluated between week 6 and 4 months). • Primary care costs for CG: USD 189.39. • Higher medication costs for the LG (evaluated before 4 months).	The average cost of laser sessions may be overestimated since only 28 of 37 patients completed all 18 sessions.
Lopes Matins et al. [32]	USD	Average cost of laser sessions per patient: USD 900.16 (34 sessions). • Additional interventions in CG: USD 185.17 (average cost). • Incremental cost of laser therapy in the LG: USD 857.3.	PBM was more cost-effective in: • Preventing severe OM. • Reducing the impact on oral health-related quality of life. • Reducing the need for RT interruptions.
Antunes et al. [33]	USD	Additional costs in the CG (without laser therapy): USD 251.15. • Incremental cost of laser therapy in the LG: USD 1689 per patient. • Incremental cost in the CG (without laser therapy) was higher at USD 251.14, due to OM-related toxicity and symptoms.	PBM had a greater impact on the economic cost of treatment but reduced the incidence of OM, as well as additional costs associated with it.

Abbreviations: LG (laser group), CG (control group), OM (oral mucositis), RT (radiotherapy), and PBM (photobiomodulation).

**Table 4:** Analysis of the certainty of evidence.

Certainty Assessment							№ of Patients		Effect		Certainty	Importance
№ of Studies	Study Design	Risk of Bias	Inconsistency	Indirect Evidence	Imprecision	Other Considerations	PBM	P	Relative (95% CI)	Absolute (95% CI)		
**Cost-effectiveness analysis**												
3	Randomized clinical trials	Not serious	Not serious	Not serious	Serious ^a^	None	116	113	Not estimable		⨁⨁⨁◯ Moderate ^a^	CRITICAL
**Reduction in severe oral mucositis incidence**												
3	Randomized clinical trials	Not serious	Not serious	Not serious	Serious ^b^	None	116	113	Not estimable		⨁⨁⨁◯ Moderate ^b^	CRITICAL

Abbreviations: CI (Confidence interval), PBM (photobiomodulation group), and P (placebo group). Explanations: ^a^ The studies presented a certain degree of imprecision primarily due to the relatively small sample sizes. ^b^ Small sample number, ⨁⨁⨁◯: Moderate certainty.

## Data Availability

The datasets generated during and/or analyzed during the current study are available from the corresponding author on reasonable request.

## Supplementary Materials

The following supporting information can be downloaded at: https://www.mdpi.com/article/10.3390/biomedicines12102366/s1, Supplementary Materials S1—Search Strategy of the Study.
