# Supplementary material for: Assessing the Cost-Effectiveness of Photobiomodulation for Oral Mucositis Prevention and Treatment: A Systematic Review
Source: Biomedicines. 2024 Oct 16;12(10):2366. doi: 10.3390/biomedicines12102366 (PMC11505555; doi:10.3390/biomedicines12102366)
Supplement: Supplementary file 1 [file biomedicines-12-02366-s001.zip › biomedicines-3258048-supplementary.pdf]

Supplementary Materials S1. Database Search strategy: July 23, 2024

Database	Search
Pubmed<br>8	(“Light Therapies, Low-Level” OR “Light Therapy, Low-Level” OR “Low Level Light Therapy” OR “Low-Level Light Therapies” OR “Therapies, Low-Level Light” OR “Therapy, Low-Level Light” OR “Photobiomodulation Therapy” OR “Photobiomodulation Therapies” OR “Therapies, Photobiomodulation” OR “Therapy, Photobiomodulation” OR “Photobiomodulation” OR “Photobiomodulations” OR “LLLT” OR “Laser Therapy, Low-Level” OR “Laser Therapies, Low- Level” OR “Laser Therapy, Low Level” OR “Low-Level Laser Therapies” OR “Laser Irradiation, Low-Power” OR “Irradiation, Low-Power Laser” OR “Laser Irradiation, Low Power” OR “Low- Power Laser Therapy” OR “Low Power Laser Therapy” OR “Laser Therapy, Low-Power” OR “Laser Therapies, Low-Power” OR “Laser Therapy, Low Power” OR “Low-Power Laser Therapies” OR “Low-Level Laser Therapy” OR “Low Level Laser Therapy” OR “Low-Power Laser Irradiation” OR “Low Power Laser Irradiation” OR “Laser Biostimulation” OR “Biostimulation, Laser” OR “Laser Phototherapy Phototherapy, Laser” OR “Low-Level Light Therapy”) AND (Stomatitis OR “Stomatitides” OR “Oral Mucositis” OR “Mucositides, Oral” OR “Oral Mucositides” OR “Oromucositis” OR “Oromucositides” OR “Mucositis, Oral”) AND (“Cost- Effectiveness Analysis” OR “Analysis, Cost-Effectiveness” OR “Cost Effectiveness Analysis” OR “Cost Effectiveness” OR “Effectiveness, Cost” OR “Cost Effectiveness Ratio” OR “Cost Effectiveness Ratios” OR “Effectiveness Ratio, Cost” OR “Ratio, Cost Effectiveness”)
EMBASE<br>21	('light therapies, low-level' OR 'light therapy, low-level' OR 'low level light therapy'/exp OR 'low level light therapy' OR 'low-level light therapies' OR 'therapies, low-level light' OR 'therapy, low-level light' OR 'photobiomodulation therapy'/exp OR 'photobiomodulation therapy' OR 'photobiomodulation therapies' OR 'therapies, photobiomodulation' OR 'therapy, photobiomodulation' OR 'photobiomodulation'/exp OR 'photobiomodulation' OR 'photobiomodulations' OR 'lllt' OR 'laser therapy, low-level'/exp OR 'laser therapy, low-level' OR 'laser therapies, low- level' OR 'laser therapy, low level'/exp OR 'laser therapy, low level' OR 'low-level laser therapies' OR 'laser irradiation, low-power' OR 'irradiation, low-power laser' OR 'laser irradiation, low power' OR 'low- power laser therapy'/exp OR 'low- power laser therapy' OR 'low power laser therapy'/exp OR 'low power laser therapy' OR 'laser therapy, low-power' OR 'laser therapies, low-power' OR 'laser therapy, low power' OR 'low-power laser therapies' OR 'low-level laser therapy'/exp OR 'low-level laser therapy' OR 'low level laser therapy'/exp OR 'low level laser therapy' OR 'low-power laser irradiation' OR 'low power laser irradiation'/exp OR 'low power laser irradiation' OR 'laser biostimulation'/exp OR 'laser biostimulation' OR 'biostimulation, laser' OR 'laser phototherapy phototherapy, laser' OR 'low-level light therapy'/exp OR 'low-level light therapy') AND ('stomatitis'/exp OR stomatitis OR 'stomatitides' OR 'oral mucositis'/exp OR 'oral mucositis' OR 'mucositides, oral' OR 'oral mucositides'/exp OR 'oral mucositides' OR 'oromucositis'/exp OR 'oromucositis' OR 'oromucositides'/exp OR 'oromucositides' OR 'mucositis, oral') AND ('cost- effectiveness analysis'/exp OR 'cost- effectiveness analysis' OR 'analysis, cost-effectiveness' OR 'cost effectiveness analysis'/exp OR 'cost effectiveness analysis' OR 'cost effectiveness'/exp OR 'cost effectiveness' OR 'effectiveness, cost' OR 'cost effectiveness ratio'/exp OR 'cost effectiveness ratio' OR 'cost effectiveness ratios' OR 'effectiveness ratio, cost' OR 'ratio, cost effectiveness')
Web of Science<br>10	TS=((“Light Therapies, Low-Level” OR “Light Therapy, Low-Level” OR “Low Level Light Therapy” OR “Low-Level Light Therapies” OR “Therapies, Low-Level Light” OR “Therapy, Low-<br>Level Light” OR “Photobiomodulation Therapy” OR “Photobiomodulation Therapies” OR “Therapies, Photobiomodulation” OR “Therapy, Photobiomodulation” OR “Photobiomodulation” OR “photobiomodulation” OR “LLLT” OR “Laser Therapy, Low-Level” OR “Laser Therapies, Low-Level” OR “Laser Therapy, Low Level” OR “Low-Level Laser Therapies” OR “Laser Irradiation, Low-Power” OR “Irradiation, Low-Power Laser” OR “Laser Irradiation, Low Power” OR “Low-Power Laser Therapy” OR “Low Power

	Laser Therapy" OR "Laser Therapy, Low- Power" OR "Laser Therapies, Low-Power" OR "Laser Therapy, Low Power" OR "Low-Power Laser Therapies" OR "Low-Level Laser Therapy" OR "Low Level Laser Therapy" OR "Low- Power Laser Irradiation" OR "Low Power Laser Irradiation" OR "Laser Biostimulation" OR "Biostimulation, Laser" OR "Laser Phototherapy Phototherapy, Laser" OR "Low-Level Light Therapy") AND (Stomatitis OR "stomatitiden" OR "Oral Mucositis" OR "Mucositides, Oral" OR "Oral Mucositides" OR "oralmucositis" OR "oromucositis" OR "Mucositis, Oral") AND ("Cost-Effectiveness Analysis" OR "Analysis, Cost-Effectiveness" OR "Cost Effectiveness Analysis" OR "Cost Effectiveness" OR "Effectiveness, Cost" OR "Cost Effectiveness Ratio" OR "Cost
SCOPUS<br>164	ALL (("Light Therapies, Low-Level" OR "Light Therapy, Low-Level" OR "Low Level Light Therapy" OR "Low-Level Light Therapies" OR "Therapies, Low-Level Light" OR "Therapy, Low-Level Light" OR "Photobiomodulation Therapy" OR "Photobiomodulation Therapies" OR "Therapies, Photobiomodulation" OR "Therapy, Photobiomodulation" OR "Photobiomodulation" OR "Photobiomodulations" OR "LLLT" OR "Laser Therapy, Low-Level" OR "Laser Therapies, Low- Level" OR "Laser Therapy, Low Level" OR "Low-Level Laser Therapies" OR "Laser Irradiation, Low-Power" OR "Irradiation, Low-Power Laser" OR "Laser Irradiation, Low Power" OR "Low- Power Laser Therapy" OR "Low Power Laser Therapy" OR "Laser Therapy, Low-Power" OR "Laser Therapies, Low-Power" OR "Laser Therapy, Low Power" OR "Low-Power Laser Therapies" OR "Low-Level Laser Therapy" OR "Low Level Laser Therapy" OR "Low-Power Laser Irradiation" OR "Low Power Laser Irradiation" OR "Laser Biostimulation" OR "Biostimulation, Laser" OR "Laser Phototherapy Phototherapy, Laser" OR "Low-Level Light Therapy") AND (stomatitis OR "Stomatitides" OR "Oral Mucositis" OR "Mucositides, Oral" OR "Oral Mucositides" OR "Oromucositis" OR "Oromucositides" OR "Mucositis, Oral") AND ("Cost- Effectiveness Analysis" OR "Analysis, Cost-Effectiveness" OR "Cost Effectiveness Analysis" OR "Cost Effectiveness" OR "Effectiveness, Cost" OR "Cost Effectiveness Ratio" OR "Cost Effectiveness Ratios" OR "Effectiveness Ratio, Cost" OR "Ratio, Cost Effectiveness"))
OpenGrey<br>45	("Light Therapies, Low-Level" OR "Light Therapy, Low-Level" OR "Low Level Light Therapy" OR "Low-Level Light Therapies" OR "Therapies, Low-Level Light" OR "Therapy, Low-Level Light" OR "Photobiomodulation Therapy" OR "Photobiomodulation Therapies" OR "Therapies, Photobiomodulation" OR "Therapy, Photobiomodulation" OR "Photobiomodulation" OR "Photobiomodulations" OR "LLLT" OR "Laser Therapy, Low-Level" OR "Laser Therapies, Low- Level" OR "Laser Therapy, Low Level" OR "Low-Level Laser Therapies" OR "Laser Irradiation, Low-Power" OR "Irradiation, Low-Power Laser" OR "Laser Irradiation, Low Power" OR "Low- Power Laser Therapy" OR "Low Power Laser Therapy" OR "Laser Therapy, Low-Power" OR "Laser Therapies, Low-Power" OR "Laser Therapy, Low Power" OR "Low-Power Laser Therapies" OR "Low-Level Laser Therapy" OR "Low Level Laser Therapy" OR "Low-Power Laser Irradiation" OR "Low Power Laser Irradiation" OR "Laser Biostimulation" OR "Biostimulation, Laser" OR "Laser Phototherapy Phototherapy, Laser" OR "Low-Level Light Therapy") AND (Stomatitis OR "Stomatitides" OR "Oral Mucositis" OR "Mucositides, Oral" OR "Oral Mucositides" OR "Oromucositis" OR "Oromucositides" OR "Mucositis, Oral") AND ("Cost- Effectiveness Analysis" OR "Analysis, Cost-Effectiveness" OR "Cost Effectiveness Analysis" OR "Cost Effectiveness" OR "Effectiveness, Cost" OR "Cost Effectiveness Ratio" OR "Cost Effectiveness Ratios" OR "Effectiveness Ratio, Cost" OR "Ratio, Cost Effectiveness")
